# Supplementary material for: GTalign: spatial index-driven protein structure alignment, superposition, and search
Source: Nat Commun. 2024 Aug 24;15:7305. doi: 10.1038/s41467-024-51669-z (PMC11344802; doi:10.1038/s41467-024-51669-z)
Supplement: Supplementary file 1 — Supplementary Information [file 41467_2024_51669_MOESM1_ESM.pdf]

Supplementary Information for: GTalign: Spatial index-driven protein structure alignment, superposition, and search

Mindaugas Margelevičius[1*]

[1*]Institute of Biotechnology, Life Sciences Center, Vilnius University, Vilnius, Lithuania.

Corresponding author(s). E-mail(s): mindaugas.margelevicius@bti.vu.lt;

Contents

S1 Supplementary results **2**
- S1.1 Reference-free performance evaluation . . . 2
  - S1.1.1 Supplementary tables . . . 2
  - S1.1.2 Supplementary figures . . . 5
- S1.2 GTalign runtimes on different machines . . . 10
- S1.3 Alignment evaluation using RMSD . . . 11
- S1.4 Benchmarking against SCOPe reference . . . 13
- S1.5 Alignment of large complexes . . . 16
- S1.6 Clustering protein structures with GTalign . . . 17

S2 Supplementary methods **18**
- S2.1 Extensive superposition search . . . 18

Bibliography **23**

S1 Supplementary results

S1.1 Reference-free performance evaluation

S1.1.1 Supplementary tables.

Table S1 Runtimes and cumulative TM-score, normalized by the length of the shorter protein, for each dataset. Additionally, runtimes are provided for GTalign evaluated on one, two, and three Tesla V100 GPUs. Numbers in parentheses indicate the maximum number of top hits evaluated.

	SCOPe40 2.08 (750K)		*PDB20* (112K)		*Swiss-Prot* (312K)	
	Runtime (*Seconds*)	Cumulative TM-score	Runtime (*Seconds*)	Cumulative TM-score	Runtime (*Seconds*)	Cumulative TM-score
3× *Tesla V100*						
GTalign --speed=0	4476.2	418682.3	872.6	65030.2	8453.8	184532.0
GTalign --speed=9	1301.4	414564.9	223.9	64414.0	1896.3	182556.0
GTalign --speed=9 --pre-score=0.4	679.7	411984.3	113.5	64156.6	899.2	181464.4
GTalign --speed=13	592.5	407922.2	104.6	63658.3	933.2	179738.8
GTalign --speed=13 --pre-score=0.4	381.7	405980.2	69.9	63482.3	618.0	178865.1
GTalign --speed=13 --pre-similarity=15	155.2	220411.3	46.3	37321.9	428.4	124803.3
2× *Tesla V100*						
GTalign --speed=0	6458.1	418682.3	1236.6	65030.2	11417.8	184532.0
GTalign --speed=9	1872.2	414564.9	316.2	64414.0	2660.0	182556.0
GTalign --speed=9 --pre-score=0.4	953.5	411984.3	157.7	64156.6	1253.0	181464.4
GTalign --speed=13	857.6	407922.2	147.0	63658.3	1325.7	179738.8
GTalign --speed=13 --pre-score=0.4	543.1	405980.2	96.5	63482.3	896.9	178865.1
GTalign --speed=13 --pre-similarity=15	214.2	220411.3	61.3	37321.9	611.5	124803.3
1× *Tesla V100*						
GTalign --speed=0	11902.0	418682.3	2261.2	65030.2	19071.6	184532.0
GTalign --speed=9	3472.7	414564.9	585.6	64414.0	4551.6	182556.0
GTalign --speed=9 --pre-score=0.4	1680.8	411984.3	280.4	64156.6	2075.7	181464.4
GTalign --speed=13	1601.2	407922.2	272.3	63658.3	2345.3	179738.8
GTalign --speed=13 --pre-score=0.4	997.8	405980.2	177.4	63482.3	1573.1	178865.1
GTalign --speed=13 --pre-similarity=15	386.2	220411.3	108.7	37321.9	1055.0	124803.3
TM-align	61380.4	416074.0	28504.8	64870.4	879965.5	183432.0
TM-align -fast	18501.2	409503.4	4826.5	64236.8	109319.0	181464.3
DeepAlign	527135.4	395118.6	187205.0	61771.6	–	–
DALI	195576.3	329225.8	49194.4	39914.8	–	–
FATCAT	369843.5	304491.7	102395.7	43501.8	–	–
Foldseek	48.5	180361.8	19.3	24513.0	195.6	46537.8
Foldseek --tmalign-fast 1	622.8	315751.3	203.3	43699.7	1138.8	75453.9
Foldseek --tmalign-fast 0	2048.1	325148.9	1156.0	45350.8	9649.5	77717.0

Table S2 Number of top hits at different levels of TM-score, normalized by the shorter protein length, for each dataset

	# top hits with TM-score≥				
	0.5	0.6	0.7	0.8	0.9
SCOPe40 2.08					
GTalign --speed=0	732024	138508	26891	5261	579
GTalign --speed=9	657116	127698	25114	5121	571
GTalign --speed=9 --pre-score=0.4	607322	123361	24871	5107	571
GTalign --speed=13	553470	113465	23378	4966	567
GTalign --speed=13 --pre-score=0.4	525719	110522	23186	4956	567
GTalign --speed=13 --pre-similarity=15	19757	7357	3756	2085	434
TM-align	683996	131059	25466	5140	573
TM-align -fast	580689	115546	22483	4655	558
DeepAlign	413179	92809	20251	4443	477
DALI	203818	43864	9300	2850	469
FATCAT	310773	66248	13951	3474	477
Foldseek	13371	6136	3292	1410	195
Foldseek --tmalign-fast 1	135399	38086	9454	2984	486
Foldseek --tmalign-fast 0	150894	41461	9862	3001	486
PDB20					
GTalign --speed=0	111603	32860	10233	2558	308
GTalign --speed=9	101965	31051	9869	2487	293
GTalign --speed=9 --pre-score=0.4	97440	30501	9794	2483	293
GTalign --speed=13	90983	29288	9537	2418	289
GTalign --speed=13 --pre-score=0.4	88693	28916	9464	2415	289
GTalign --speed=13 --pre-similarity=15	3692	1480	651	177	30
TM-align	106869	32428	10424	2694	335
TM-align -fast	97229	30894	10010	2543	313
DeepAlign	74107	24883	8047	1921	163
DALI	19856	5908	1647	228	30
FATCAT	58644	18813	5485	1056	81
Foldseek	3018	1227	352	78	14
Foldseek --tmalign-fast 1	17066	6086	1864	270	35
Foldseek --tmalign-fast 0	18987	6539	1901	274	35
Swiss-Prot					
GTalign --speed=0	306488	116253	34008	6864	1581
GTalign --speed=9	281858	108747	32867	6850	1580
GTalign --speed=9 --pre-score=0.4	264427	106267	32784	6850	1580
GTalign --speed=13	250953	99489	31463	6847	1580
GTalign --speed=13 --pre-score=0.4	240320	97986	31402	6847	1580
GTalign --speed=13 --pre-similarity=15	26288	13245	5593	2403	1356
TM-align	292670	111400	33415	6895	1582
TM-align -fast	268250	105747	31686	6535	1562
Foldseek	17400	7201	2912	1622	1081
Foldseek --tmalign-fast 1	42007	25957	11303	2783	1461
Foldseek --tmalign-fast 0	43913	27242	11730	2785	1461

Table S3 Cumulative TM-score, normalized by the query length, for each dataset. Numbers in parentheses indicate the maximum number of top hits evaluated.

	SCOPe40 2.08 (520K) Cumulative TM-score	*PDB20* (30K) Cumulative TM-score	*Swiss-Prot* (100K) Cumulative TM-score
GTalign --speed=0	289050.2	16426.2	58293.0
GTalign --speed=9	286252.7	16232.2	57587.2
GTalign --speed=9 --pre-score=0.4	284808.9	16057.3	57143.3
GTalign --speed=13	281944.0	15865.4	56483.2
GTalign --speed=13 --pre-score=0.4	280877.5	15739.2	55886.1
GTalign --speed=13 --pre-similarity=15	171378.9	11707.7	44383.7
TM-align	287250.9	16293.6	57764.3
TM-align -fast	282231.7	16010.5	56964.2
DeepAlign	174382.5	10526.1	–
DALI	205760.3	12216.4	–
FATCAT	198691.8	10438.4	–
Foldseek	111670.0	7698.6	29400.7
Foldseek --tmalign-fast 1	209747.8	13246.8	43590.1
Foldseek --tmalign-fast 0	213999.1	13446.6	44166.4

Table S4 Number of top hits at different levels of TM-score, normalized by the query length, for each dataset

	# top hits with TM-score≥				
	0.5	0.6	0.7	0.8	0.9
SCOPe40 2.08					
GTalign --speed=0	492887	90869	18336	3715	359
GTalign --speed=9	442998	83972	17156	3612	354
GTalign --speed=9 --pre-score=0.4	415248	81732	17003	3601	354
GTalign --speed=13	377275	75003	15986	3505	352
GTalign --speed=13 --pre-score=0.4	361914	73491	15864	3497	352
GTalign --speed=13 --pre-similarity=15	13879	5657	3147	1662	273
TM-align	460847	86062	17332	3624	354
TM-align -fast	386777	74277	14860	3228	340
DeepAlign	284743	63018	14048	3192	312
DALI	122228	26851	6581	2155	289
FATCAT	208110	43679	9961	2582	296
Foldseek	10375	5066	2787	1062	127
Foldseek --tmalign-fast 1	85776	24238	6943	2262	297
Foldseek --tmalign-fast 0	94430	25964	7204	2271	297
PDB20					
GTalign --speed=0	22913	5024	1061	166	23
GTalign --speed=9	20369	4665	1017	166	23
GTalign --speed=9 --pre-score=0.4	18339	4415	1006	166	23
GTalign --speed=13	16426	4137	973	166	23
GTalign --speed=13 --pre-score=0.4	15339	3983	963	166	23
GTalign --speed=13 --pre-similarity=15	2261	974	412	125	22
TM-align	21209	4739	1024	167	23
TM-align -fast	17861	4341	985	166	23
DeepAlign	13086	3261	701	114	15
DALI	12341	3562	840	149	20
FATCAT	11906	3498	842	143	20
Foldseek	1894	711	226	55	7
Foldseek --tmalign-fast 1	10356	3534	949	164	23
Foldseek --tmalign-fast 0	11423	3769	964	165	23
Swiss-Prot					
GTalign --speed=0	74533	38977	14016	2482	1277
GTalign --speed=9	70619	36366	13526	2483	1276
GTalign --speed=9 --pre-score=0.4	68870	35791	13452	2483	1276
GTalign --speed=13	64699	33200	12478	2482	1276
GTalign --speed=13 --pre-score=0.4	63355	32791	12423	2482	1276
GTalign --speed=13 --pre-similarity=15	18785	10385	4906	2180	1180
TM-align	72030	36596	13679	2483	1276
TM-align -fast	67516	34336	12882	2482	1276
Foldseek	14617	6737	2665	1480	891
Foldseek --tmalign-fast 1	30238	19396	9954	2482	1277
Foldseek --tmalign-fast 0	31357	20229	10343	2483	1276

S1.1.2 Supplementary figures.

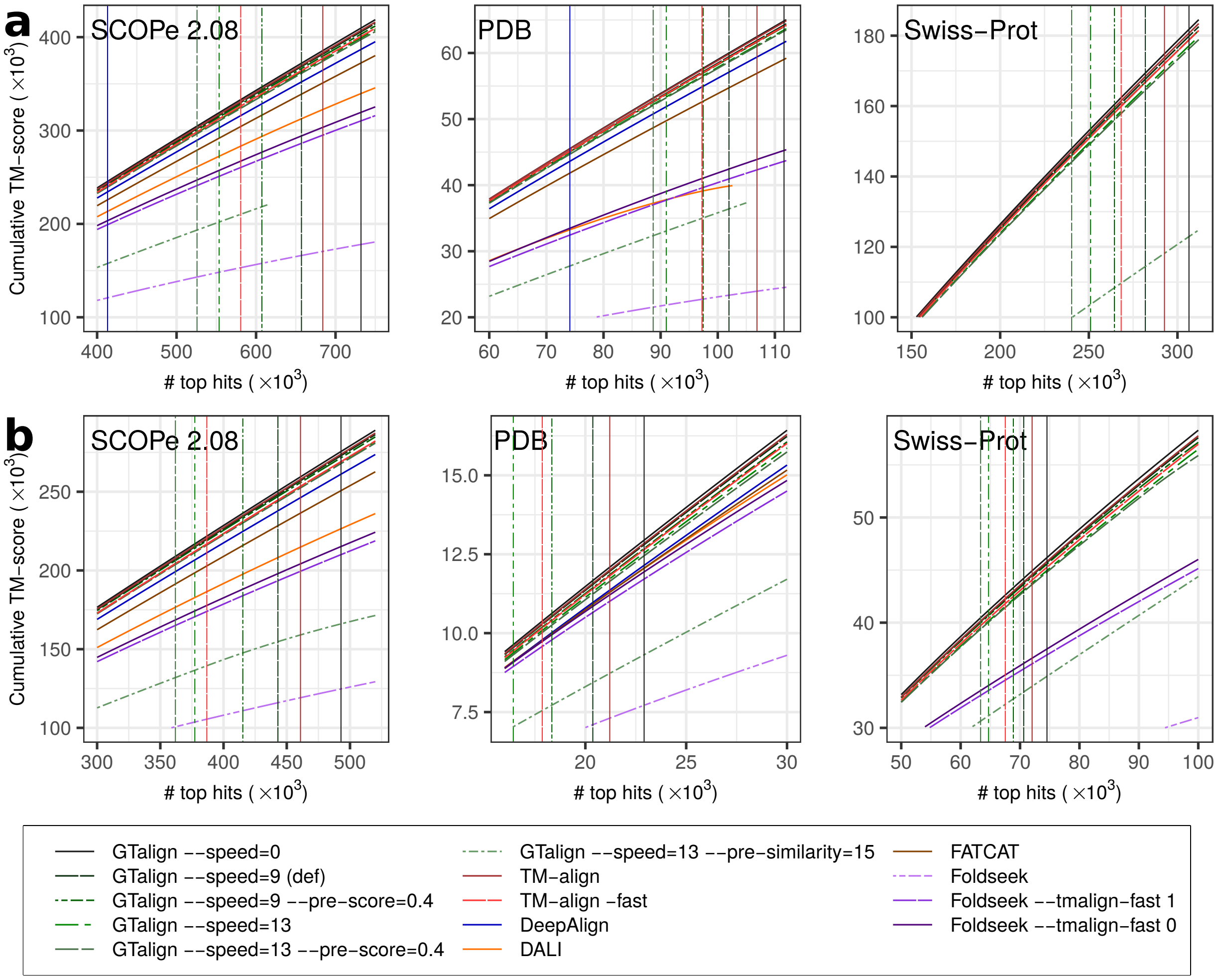

Fig. S1 Benchmarking results on the SCOPe40 2.08, PDB20, and Swiss-Prot datasets. The figure plots the cumulative TM-score against the number of top alignments sorted by TM-align-obtained TM-score and represents zoomed-in sections of Fig. 1 and 2 from the main text for detailed analysis. Vertical lines indicate the number of alignments with a TM-score ≥ 0.5. Panels (a) and (b) show TM-scores normalized by the length of the shorter protein and by the query length, respectively. Source data are provided as a Source Data file.

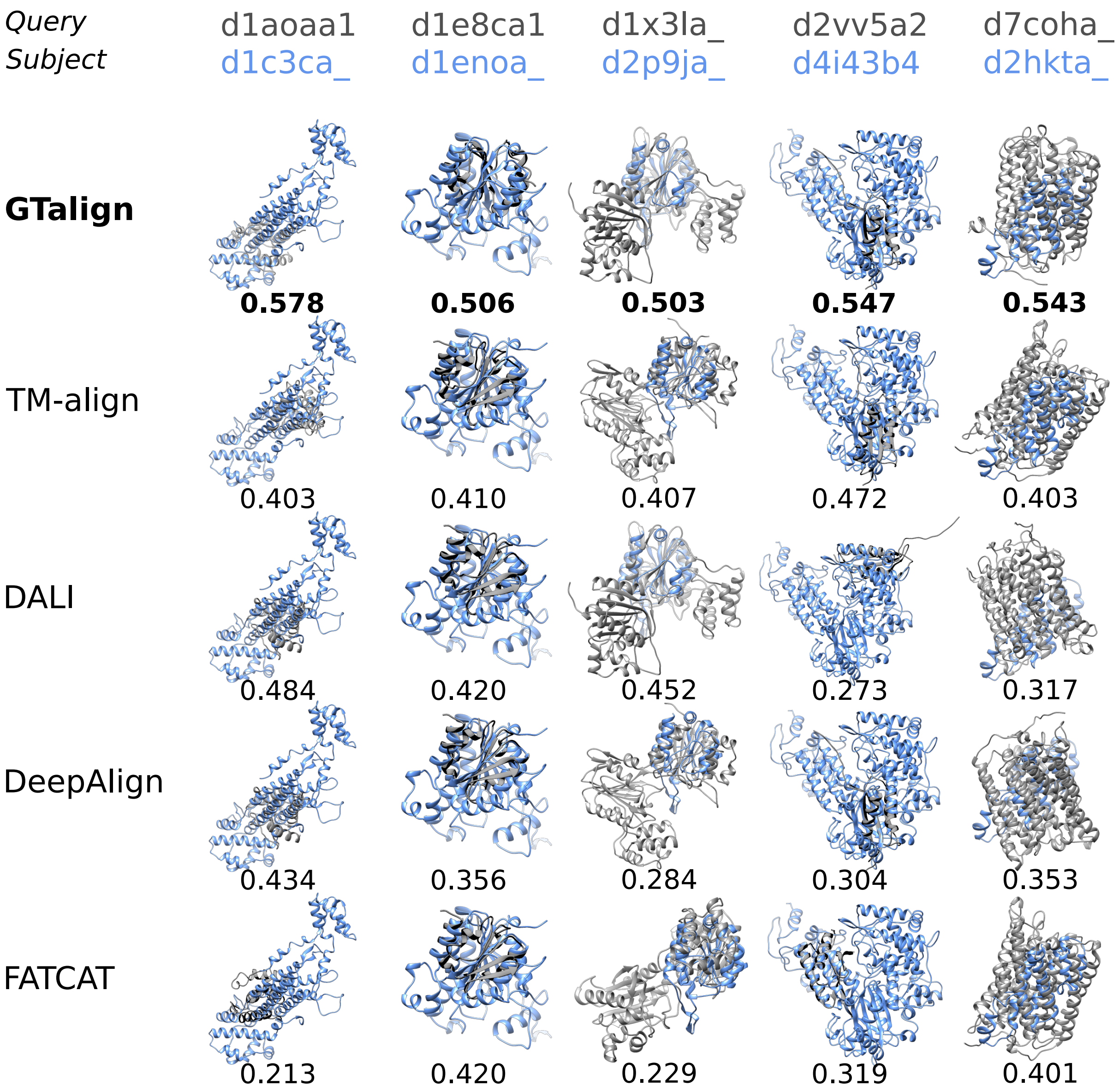

Fig. S2 Structural alignment examples from benchmarking on the SCOPe40 2.08 dataset. Query structures are in grey, and subject structures are colored. The subject structures share the same orientation across the tools for visual inspection. Numbers represent TM-scores normalized by the length of the shorter protein. It's noteworthy that some superpositions appear similar despite having different TM-scores. In such instances, TM-align identifies a favorable superposition even when provided with an inferior alignment. Foldseek (all parameterized versions) did not produce alignments for the given protein pairs.

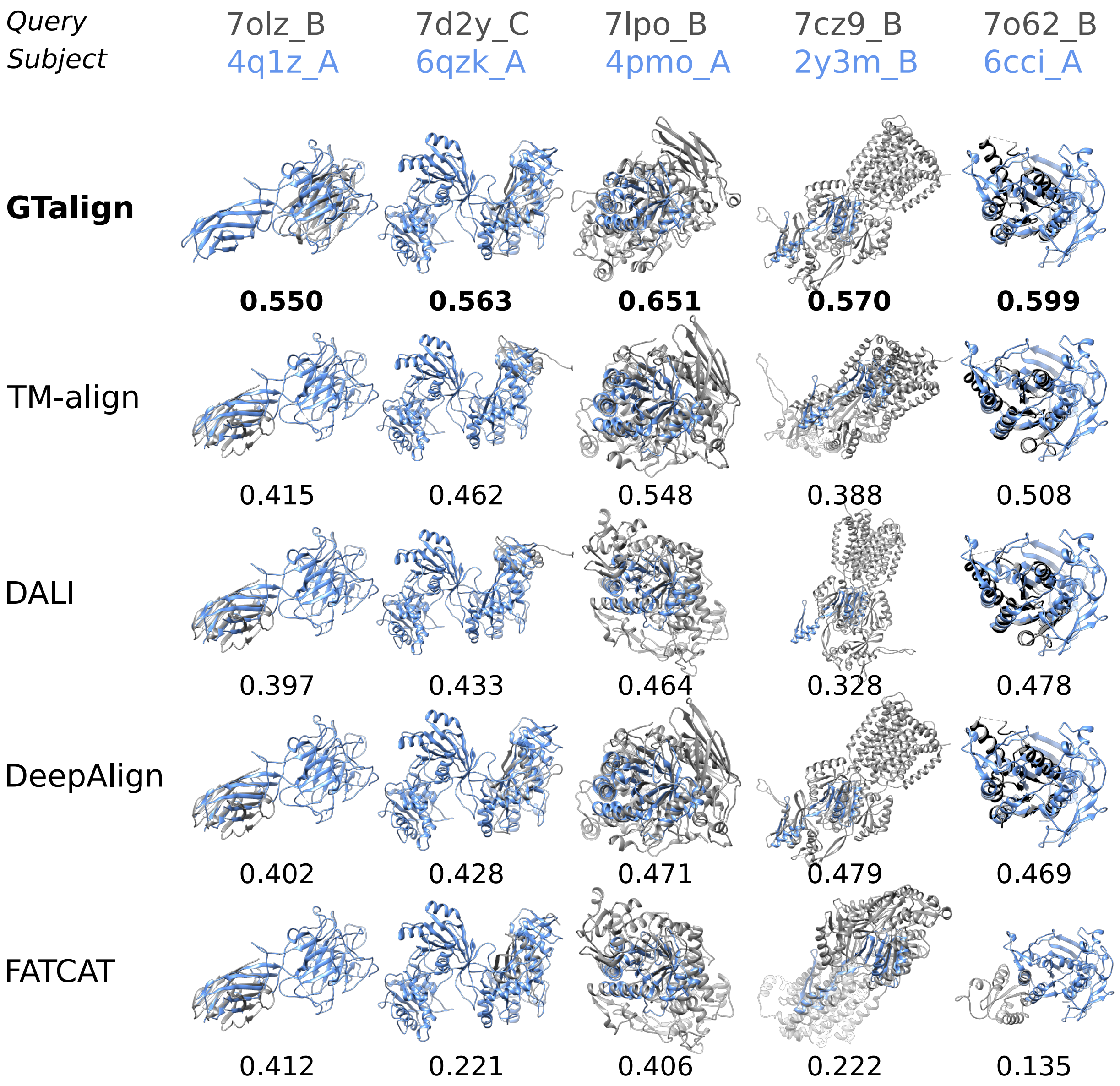

Fig. S3 Illustrative examples from benchmarking on the PDB20 dataset. Query structures are in grey, and subject structures are colored. The subject structures share the same orientation across the tools. Numbers represent TM-scores normalized by the length of the shorter protein. Foldseek (all parameterized versions) did not produce alignments for the given protein pairs.

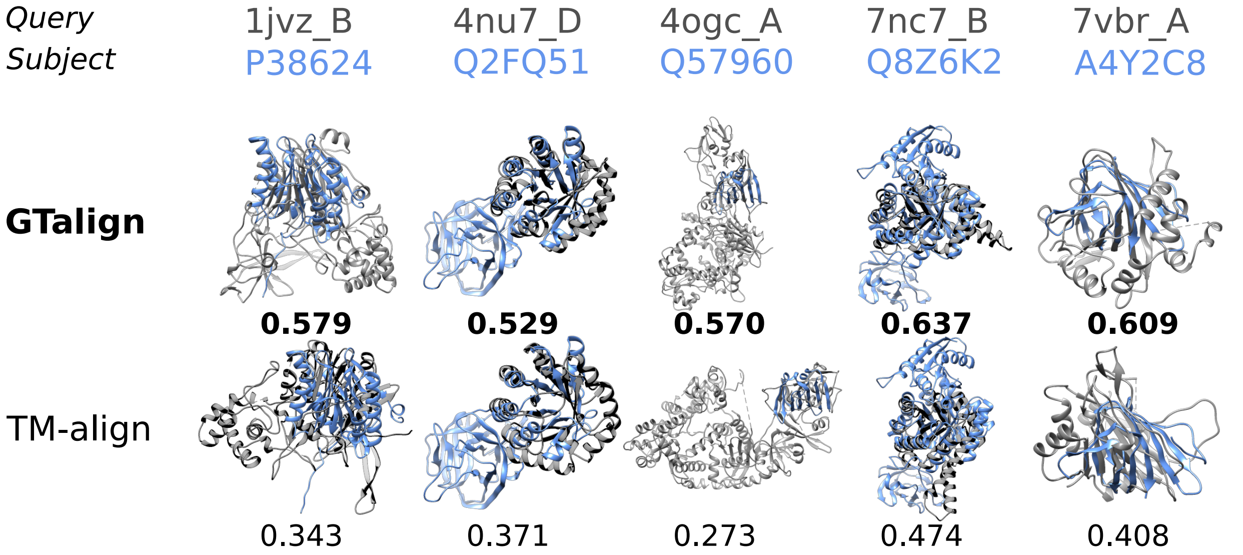

Fig. S4 Illustrative examples from benchmarking on the Swiss-Prot dataset. Query structures are in grey. Subject structures are colored. The subject structures share the same orientation across the tools. Numbers represent TM-scores normalized by the length of the shorter protein. Foldseek (all parameterized versions) did not produce alignments for the given protein pairs.

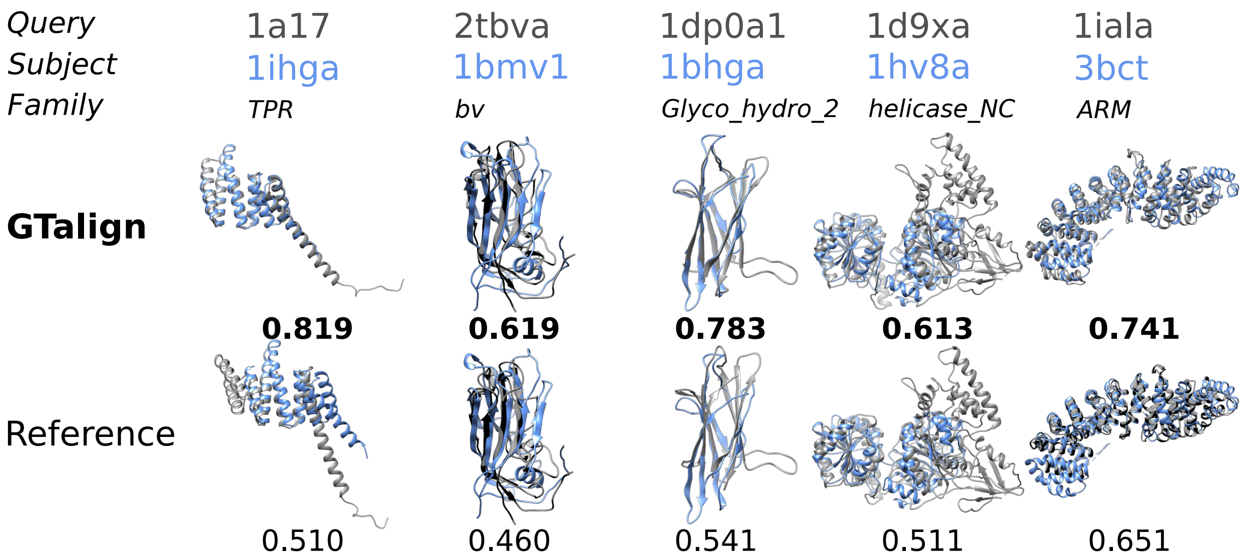

Fig. S5 Illustrative examples of GTalign and reference (original) alignments from benchmarking on the HOMSTRAD dataset. Query structures are depicted in grey, while subject structures are colored. The same subject structures maintain the same orientation for comparison. Numbers represent TM-scores normalized by the length of the shorter protein.

S1.2 GTalign runtimes on different machines

In assessing the performance of GTalign parameterized variants across diverse hardware configurations, we conducted runtime evaluations on three distinct machines: a server-grade system equipped with three Tesla V100 GPU accelerators, a desktop-grade machine featuring a GeForce RTX 4090 GPU, and a laptop with a GeForce RTX 4090 Laptop GPU. GTalign was configured to use 16GB of GPU RAM (option `--dev-mem`) for all tests.

Notably, the desktop-grade machine, housing a more recent and affordable GeForce RTX 4090 GPU, outpaced the server with three Tesla V100 GPU cards when running GTalign. The detailed runtimes for each GTalign parameterized variant on these diverse machines are presented in Table S5.

Table S5 Runtimes (in seconds) of parameterized GTalign variants on different machines for each dataset

	3×Tesla V100 (*Server*)	1×GeForce RTX 4090 (*Desktop*)	1×GeForce RTX 4090 Laptop (*Laptop*)
SCOPe40 2.08			
GTalign --speed=0	4476.2	3762.5	8522.6
GTalign --speed=9	1301.4	1082.9	2332.5
GTalign --speed=13 --pre-similarity=15	155.2	159.6	210.7
PDB20			
GTalign --speed=0	872.6	781.7	1702.3
GTalign --speed=9	223.9	193.5	383.4
GTalign --speed=13 --pre-similarity=15	46.3	40.8	55.8
Swiss-Prot			
GTalign --speed=0	8453.8	6544.2	15612.2
GTalign --speed=9	1896.3	1400.7	3080.8
GTalign --speed=13 --pre-similarity=15	428.4	371.0	607.5

S1.3 Alignment evaluation using RMSD

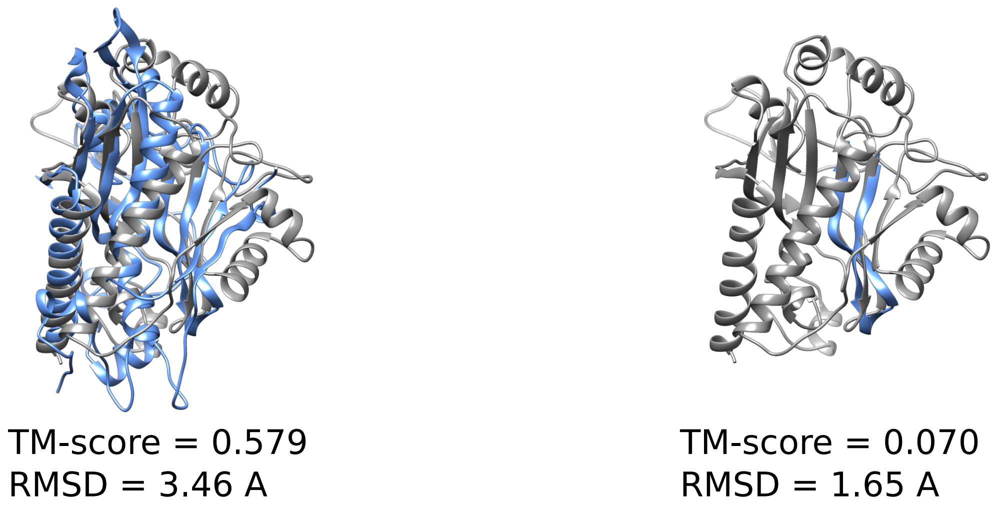

Fig. S6 Illustrative example of alignment accuracy evaluation for the SCOPe 2.08 domains d12asa_ (d.104.1.1; shown in grey) and d1seta2 (d.104.1.1; shown in color) using TM-score normalized by the length of the shorter protein and RMSD. TM-score is sensitive to alignment coverage due to normalization by protein length. RMSD, on the other hand, is normalized by the number of aligned residue pairs and measures their spatial proximity after superposition, similar to TM-score. Therefore, a short accurately aligned fragment (right panel; unaligned d1seta2 parts not shown) implies a lower RMSD compared to the full alignment (left panel), which includes more divergent structural segments. Alignments, TM-scores, and RMSDs were calculated using TM-align.

Table S6 Mean RMSDs (in angstroms), GDT_TS scores normalized by the number of aligned residues, and numbers of aligned residues (Length) for each dataset. Numbers in parentheses following the dataset names indicate the maximum number of top hits evaluated. s.d., standard deviation.

	RMSD (s.d.)	GDT_TS (s.d.)	Length (s.d.)
SCOPe40 2.08 (750K)			
GTalign --speed=0	2.91 (0.87)	0.7137 (0.1288)	67.4 (43.1)
GTalign --speed=9	2.92 (0.88)	0.7128 (0.1312)	67.0 (43.1)
GTalign --speed=9 --pre-score=0.4	2.90 (0.88)	0.7182 (0.1310)	65.0 (42.9)
GTalign --speed=13	2.92 (0.91)	0.7164 (0.1345)	65.0 (43.2)
GTalign --speed=13 --pre-score=0.4	2.90 (0.90)	0.7206 (0.1341)	63.6 (43.0)
GTalign --speed=13 --pre-similarity=15	4.87 (0.95)	0.4872 (0.1067)	94.0 (42.8)
TM-align	2.92 (0.88)	0.7144 (0.1315)	66.7 (43.3)
TM-align -fast	2.94 (0.90)	0.7121 (0.1333)	66.4 (43.5)
DeepAlign	2.48 (0.97)	0.7663 (0.1375)	56.0 (39.2)
DALI	9.49 (5.12)	0.5075 (0.1107)	109.6 (44.3)
FATCAT	4.55 (3.11)	0.6368 (0.2089)	69.7 (50.6)
Foldseek	10.26 (4.20)	0.3810 (0.1433)	85.4 (44.4)
Foldseek --tmalign-fast 1	4.37 (0.91)	0.5277 (0.0988)	96.7 (42.6)
Foldseek --tmalign-fast 0	4.31 (0.90)	0.5342 (0.0978)	98.0 (42.7)
PDB20 (112K)			
GTalign --speed=0	2.47 (1.05)	0.7781 (0.1462)	57.1 (49.0)
GTalign --speed=9	2.48 (1.06)	0.7779 (0.1479)	56.8 (48.9)
GTalign --speed=9 --pre-score=0.4	2.45 (1.05)	0.7832 (0.1453)	54.9 (47.8)
GTalign --speed=13	2.44 (1.07)	0.7852 (0.1479)	54.7 (48.6)
GTalign --speed=13 --pre-score=0.4	2.42 (1.06)	0.7887 (0.1459)	53.3 (47.5)
GTalign --speed=13 --pre-similarity=15	5.30 (1.09)	0.4436 (0.1149)	120.0 (57.5)
TM-align	2.47 (1.07)	0.7819 (0.1476)	56.3 (49.4)
TM-align -fast	2.44 (1.06)	0.7858 (0.1477)	54.9 (49.3)
DeepAlign	2.03 (1.04)	0.8285 (0.1416)	47.6 (49.0)
DALI	12.55 (7.84)	0.4587 (0.1384)	136.4 (66.3)
FATCAT	5.18 (4.32)	0.6260 (0.2565)	85.6 (78.4)
Foldseek	11.76 (5.30)	0.3501 (0.1499)	103.5 (56.2)
Foldseek --tmalign-fast 1	4.97 (1.22)	0.4714 (0.1208)	122.1 (54.3)
Foldseek --tmalign-fast 0	4.92 (1.22)	0.4777 (0.1214)	125.4 (55.4)
Swiss-Prot (312K)			
GTalign --speed=0	2.96 (1.16)	0.7113 (0.1673)	103.6 (88.8)
GTalign --speed=9	2.97 (1.18)	0.7125 (0.1706)	101.9 (88.4)
GTalign --speed=9 --pre-score=0.4	2.93 (1.18)	0.7190 (0.1706)	99.1 (88.5)
GTalign --speed=13	2.94 (1.21)	0.7186 (0.1740)	98.0 (87.5)
GTalign --speed=13 --pre-score=0.4	2.91 (1.21)	0.7238 (0.1734)	95.7 (87.3)
GTalign --speed=13 --pre-similarity=15	5.34 (1.02)	0.4365 (0.1092)	154.7 (64.4)
TM-align	2.98 (1.19)	0.7126 (0.1711)	102.7 (89.2)
TM-align -fast	2.96 (1.19)	0.7155 (0.1722)	100.1 (88.4)
DeepAlign	–	–	–
DALI	–	–	–
FATCAT	–	–	–
Foldseek	13.78 (6.49)	0.3176 (0.1563)	157.5 (82.8)
Foldseek --tmalign-fast 1	5.34 (1.29)	0.4390 (0.1197)	165.6 (68.7)
Foldseek --tmalign-fast 0	5.31 (1.28)	0.4421 (0.1193)	169.9 (68.7)

S1.4 Benchmarking against SCOPe reference

Table S7 Area under the weighted precision-recall curve (AUPRC) at the family, superfamily, and fold level. The results are presented in two parts. In one part, false positives for calculating precision and recall correspond to pairs of structures from different SCOPe 2.08 folds (No exceptions). In the second part, false positives are pairs from different SCOPe 2.08 folds; however, these pairs are ignored if the structures belong to Rossman-like (c.2–c.5, c.27, c.28, c.30, and c.31) or beta-propeller (b.66–b.70) folds [1] (Cross-fold relationships ignored).

	Cross-fold relationships ignored			*No exceptions*		
	Family	Superfamily	Fold	Family	Superfamily	Fold
GTalign --speed=0	0.807	0.591	0.0582	0.802	0.584	0.0536
GTalign --speed=9	0.808	0.592	0.0574	0.803	0.585	0.0530
GTalign --speed=9 --pre-score=0.4	0.806	0.587	0.0554	0.800	0.581	0.0509
GTalign --speed=13	0.811	0.596	0.0567	0.806	0.589	0.0521
GTalign --speed=13 --pre-score=0.4	0.808	0.590	0.0548	0.803	0.583	0.0503
GTalign --speed=13 --pre-similarity=15	0.527	0.364	0.0090	0.524	0.360	0.0069
TM-align	0.804	0.593	0.0514	0.800	0.588	0.0486
TM-align -fast	0.805	0.595	0.0505	0.801	0.590	0.0477
DeepAlign	0.669	0.540	0.0487	0.611	0.471	0.0195
DALI	0.708	0.567	0.0586	0.672	0.523	0.0339
FATCAT	0.603	0.366	0.0123	0.603	0.365	0.0123
Foldseek	0.710	0.548	0.0201	0.682	0.514	0.0109
Foldseek --tmalign-fast 1	0.733	0.523	0.0191	0.696	0.480	0.0134
Foldseek --tmalign-fast 0	0.726	0.515	0.0183	0.688	0.471	0.0129

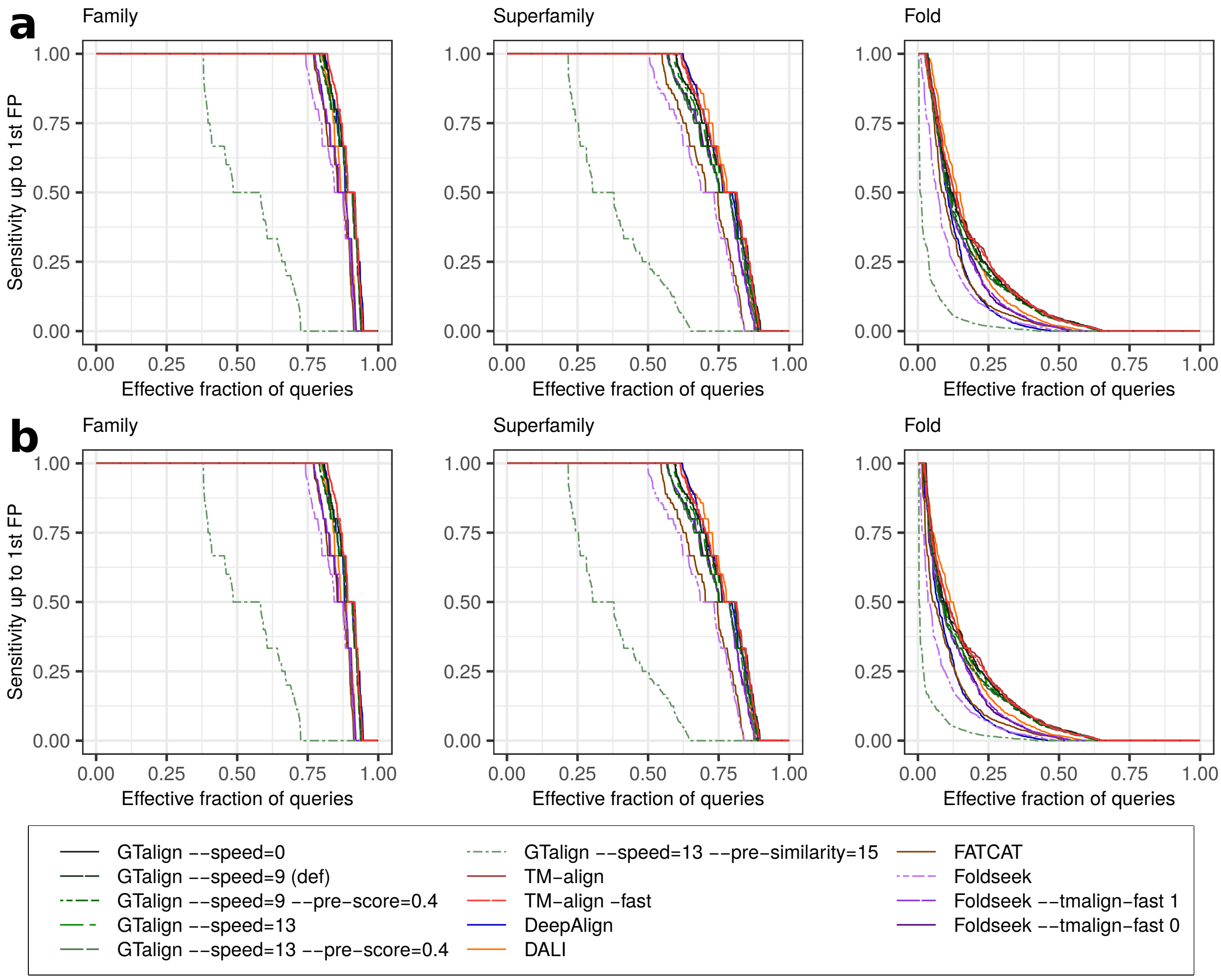

Fig. S7 Sensitivity up to the first false positive against the effective fraction of queries at the family, superfamily, and fold level. Sensitivity represents the fraction of true positives identified before encountering the first false positive. The effective fractions of queries refer to the proportion of queries originating from families with at least two members (953 queries), superfamilies with at least two families (858 queries), and folds with at least two superfamilies (941 queries), respectively. a, False positives are pairs from different SCOPe 2.08 folds, excluding those pairs belonging to Rossman-like (c.2–c.5, c.27, c.28, c.30, and c.31) or beta-propeller (b.66–b.70) folds. b, False positives are pairs from different SCOPe 2.08 folds without exceptions. Source data are provided as a Source Data file.

Table S8 Average sensitivity up to the first false positive at the family, superfamily, and fold levels. Sensitivity represents the fraction of true positives identified before the first false positive is encountered. In one analysis, false positives are defined as pairs from different SCOPe 2.08 folds, ignoring those pairs from Rossman-like (c.2–c.5, c.27, c.28, c.30, and c.31) or beta-propeller (b.66–b.70) folds (Cross-fold relationships ignored). In the second analysis, false positives are defined as pairs from different SCOPe 2.08 folds (No exceptions). P-values in parentheses are obtained from the nonparametric unpaired two-sided Mann-Whitney test on the sensitivity values (Fig. S7) between GTalign --speed=0 and all other parameterized tools at the family, superfamily, and fold levels.

	Family (p-value)	Superfamily (p-value)	Fold (p-value)
Cross-fold relationships ignored			
GTalign --speed=0	0.8933 (–)	0.7723 (–)	0.1750 (–)
GTalign --speed=9	0.8928 (0.920)	0.7723 (0.951)	0.1718 (0.853)
GTalign --speed=9 --pre-score=0.4	0.8859 (0.409)	0.7541 (0.257)	0.1622 (0.380)
GTalign --speed=13	0.8912 (0.823)	0.7664 (0.756)	0.1676 (0.569)
GTalign --speed=13 --pre-score=0.4	0.8844 (0.392)	0.7497 (0.182)	0.1595 (0.242)
GTalign --speed=13 --pre-similarity=15	0.5380 (1.1×10^{-87})	0.3752 (8.4×10^{-79})	0.0327 (1.1×10^{-52})
TM-align	0.8958 (0.558)	0.7782 (0.525)	0.1802 (0.931)
TM-align -fast	0.8966 (0.547)	0.7767 (0.577)	0.1759 (0.854)
DeepAlign	0.8908 (0.922)	0.7737 (0.498)	0.1258 (1.6×10^{-17})
DALI	0.8722 (0.556)	0.7792 (0.105)	0.1682 (0.031)
FATCAT	0.8556 (0.022)	0.7098 (0.003)	0.1219 (6.9×10^{-11})
Foldseek	0.8457 (0.003)	0.6916 (2.3×10^{-5})	0.0973 (5.0×10^{-15})
Foldseek --tmalign-fast 1	0.8616 (0.175)	0.7481 (0.263)	0.1431 (4.7×10^{-5})
Foldseek --tmalign-fast 0	0.8622 (0.193)	0.7496 (0.258)	0.1428 (2.9×10^{-5})
No exceptions			
GTalign --speed=0	0.8923 (–)	0.7705 (–)	0.1580 (–)
GTalign --speed=9	0.8918 (0.920)	0.7706 (0.952)	0.1547 (0.814)
GTalign --speed=9 --pre-score=0.4	0.8849 (0.410)	0.7526 (0.261)	0.1453 (0.347)
GTalign --speed=13	0.8902 (0.823)	0.7651 (0.810)	0.1509 (0.525)
GTalign --speed=13 --pre-score=0.4	0.8834 (0.393)	0.7487 (0.208)	0.1429 (0.212)
GTalign --speed=13 --pre-similarity=15	0.5380 (4.1×10^{-87})	0.3747 (4.8×10^{-78})	0.0261 (4.9×10^{-54})
TM-align	0.8954 (0.520)	0.7765 (0.522)	0.1630 (0.938)
TM-align -fast	0.8962 (0.510)	0.7752 (0.553)	0.1587 (0.844)
DeepAlign	0.8889 (0.973)	0.7717 (0.487)	0.1001 (5.2×10^{-20})
DALI	0.8717 (0.551)	0.7777 (0.099)	0.1486 (0.029)
FATCAT	0.8554 (0.026)	0.7082 (0.003)	0.0980 (2.2×10^{-12})
Foldseek	0.8451 (0.003)	0.6897 (1.8×10^{-5})	0.0749 (2.0×10^{-18})
Foldseek --tmalign-fast 1	0.8608 (0.163)	0.7470 (0.298)	0.1253 (3.2×10^{-5})
Foldseek --tmalign-fast 0	0.8605 (0.161)	0.7485 (0.293)	0.1242 (1.8×10^{-5})

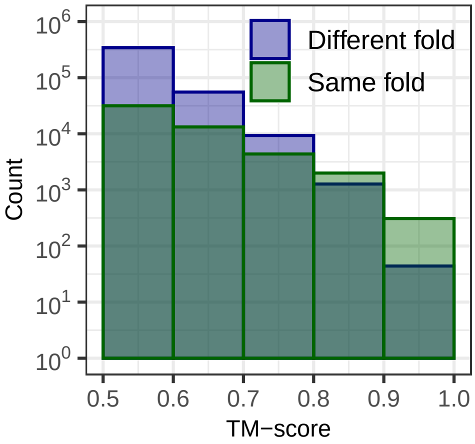

Fig. S8 Distributions of TM-scores (> 0.5) for domain pairs within the same and different SCOPe 2.08 folds, presented in bins of width 0.1. TM-scores, normalized by the query length, were calculated by aligning query and subject structures from the SCOPe40 2.08 dataset using TM-align. Source data are provided as a Source Data file.

S1.5 Alignment of large complexes

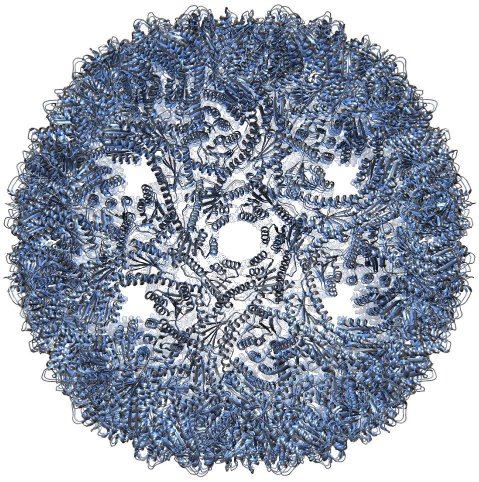

	TM-score	Runtime (sec)
GTalign --speed=13	0.2448	1.6
GTalign --speed=9	0.2793	8.7
GTalign --speed=6	0.3154	23.5
TM-align	0.3154	8,084,903.5

Fig. S9 Superposition of two virus nucleocapsid variants, 7a4i and 7a4j (37,860 residues each), by GTalign and TM-align. The GTalign runtimes exclude GPU initialization time (3.5 seconds). The GTalign command line included the following options for aligning the complexes: `--dev-queries-per-chunk=1 --dev-queries-total-length-per-chunk=40000 --dev-max-length=40000 --pre-score=0 --ter=0 -s 0`.

S1.6 Clustering protein structures with GTalign

To showcase GTalign's clustering capabilities, we performed a clustering analysis on the entire PDB [2] database (accessed 08/18/2023) of protein structures. Using GTalign with parameters `--dev-queries-total-length-per-chunk=1500 --dev-min-length=3 --dev-max-length=1500 --speed=13 --add-search-by-ss --cls-coverage=0.7 --cls-threshold=0.5 -c cachedir` and the complete linkage clustering algorithm [3] (single linkage clustering is available too), we clustered protein structures at a TM-score threshold of 0.5 with a length coverage threshold of 70%. The clustering focused solely on the first protein chains, totaling 199,839 chains, and was accomplished in five days, leveraging three Tesla V100 GPUs. The distribution of cluster sizes resulting from this analysis is visualized in Fig. S10a.

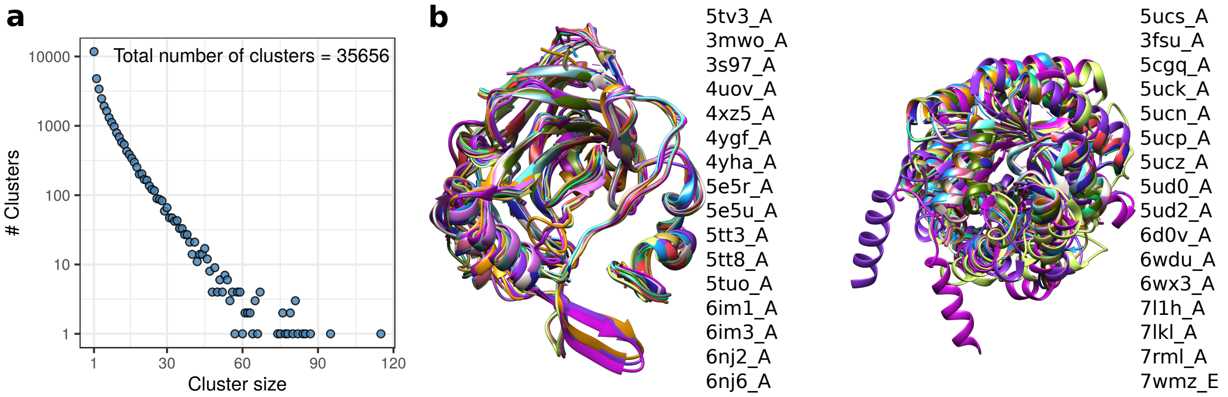

Fig. S10 Results of the GTalign clustering analysis on the entire PDB database. a, Distribution of cluster sizes. b, Superimpositions of proteins within selected clusters, representing the Carbonic anhydrase fold (left panel) and the TIM beta/alpha-barrel fold (right panel). Despite variations in sequence identity (21–35% with the first member of the first cluster and at most 7% for the second cluster, excluding trivial matches), GTalign reveals significant structural similarity within clusters. Superpositions were found by aligning the first member of each cluster with the rest using GTalign and the option `--referenced`, producing transformation matrices for subject structures. Source data are provided as a Source Data file.

The significance of clustering in the structural space becomes evident when compared to traditional sequence-based clustering approaches. As demonstrated in Fig. S10b, the superposition of cluster member proteins showcases known structural relationships also detected by GTalign. In one example, a cluster encompassing proteins with the TIM beta/alpha-barrel fold reveals diversity in sequence yet high structural similarity. More than half of the proteins within this cluster share, at most, 7% sequence identity with the cluster's first member. Despite this low sequence identity, their structural alignment yields significant TM-scores ranging from 0.553 to 0.708. In contrast, conventional sequence-based clustering would consider such protein pairs as unrelated due to the lack of sequence similarity. This example underscores the effectiveness and utility of fast clustering in the structural space, a feature offered by GTalign.

S2 Supplementary methods

S2.1 Extensive superposition search

This section presents algorithms for an extensive superposition search using spatial indices. The following notation is adopted: vectors are denoted by bold lowercase letters, and multi-dimensional matrices are represented by italic uppercase letters. The operator $[\cdot]_{\mathrm{mut}}$ in Algorithms 2 and 8 signifies a mutually exclusive operation that ensures no two threads access the same data simultaneously.

The top-level algorithm is given in Algorithm 1. It has the following parameters: n_{Q}, n_{S}, $\mathbf{l}$, $\tilde{\mathbf{l}}$, l_{f}, C, $\tilde{C}$, T, $\tilde{T}$, n_{brn}, and c_{thr}. n_{Q} and n_{S} represent the numbers of query and subject structures in their respective batches, while $\mathbf{l}$ and $\tilde{\mathbf{l}}$ denote the lengths of the query and subject structures, respectively. l_{f} represents the length of a protein sequence fragment used for initial protein superpositions within the scope of the algorithm. C and $\tilde{C}$ are the coordinates of the query and subject structures, with $C_{q,p_q} \in \mathbb{R}^{3\times1}$ indicating the coordinates of residue p_q of query structure q and $\tilde{C}_{s,\tilde{p}_s} \in \mathbb{R}^{3\times1}$ having the corresponding meaning for subject structure s. T and $\tilde{T}$ represent the secondary structure assignments for the query and subject structures. n_{brn} is a configurable number of top-performing superposition branches to explore in detail, and c_{thr} is a local structure similarity threshold for triggering superposition analysis initiated by a specific configuration of continuous fragment matching.

The algorithm initially computes the dynamic programming (DP) matrix of local scores using the following recursive equation:

$$L_{qsij} = \max\{L_{q,s,i-1,j-1} + 2 \cdot \mathbf{1}_{T_{qi}=\tilde{T}_{sj}} - 1, L_{q,s,i-1,j} - 0.8, L_{q,s,i,j-1} - 0.8, 0\}, \tag{S1}$$

with the values L_{qsij} capped at a maximum value of 255 to reduce memory footprint. Subsequently, initial superpositions (lines 6–7) are determined based on protein regions exhibiting substantial local similarity (line 5). Following the calculation of initial superpositions, several rounds of constant-time alignment, coupled with superposition inference, are executed (lines 8–13). These steps enable the rapid computation of numerous (n_{ext}) alignments based on different spatial configurations. However, the resulting alignments are sequence order-independent, some of which correspond to only good spatial agreement rather than similar folds, and necessitate post-processing.

Algorithm 1 Find most favorable superpositions by deep search

1: **procedure** DeepSuperpositionSearch(n_{Q}, n_{S}, $\mathbf{l}$, $\tilde{\mathbf{l}}$, l_{f}, C, $\tilde{C}$, T, $\tilde{T}$, n_{brn}, c_{thr})
2: Calculate L using Eq. (S1) in parallel $\forall\, 0 \le q < n_{\mathrm{Q}}$ and $0 \le s < n_{\mathrm{S}}$
3: Calculate n_{ext} based on $\max_q l_q$ and $\max_s \tilde{l}_s$ and depth specification
4: Set n_{tfm} to 32, 64, or 96 based on depth specification
5: CalculateLocalSimilarity(n_{Q}, n_{S}, n_{ext}, $\mathbf{l}$, $\tilde{\mathbf{l}}$, l_{f}, L, c_{thr})
6: CalculateXCovariances(n_{Q}, n_{S}, n_{ext}, l_{f}, C, $\tilde{C}$)
7: CalculateTransformations(n_{Q}, n_{S}, n_{ext}, $\mathbf{l}$, $\tilde{\mathbf{l}}$, 1)
8: AlignInConstantTime(n_{Q}, n_{S}, n_{ext}, $\mathbf{l}$, $\tilde{\mathbf{l}}$, C, $\tilde{C}$, T, $\tilde{T}$, 1)
9: CalculateXCovariancesA(n_{Q}, n_{S}, n_{ext}, $\mathbf{l}$, $\tilde{\mathbf{l}}$)
10: CalculateTransformations(n_{Q}, n_{S}, n_{ext}, $\mathbf{l}$, $\tilde{\mathbf{l}}$, 1)
11: AlignInConstantTime(n_{Q}, n_{S}, n_{ext}, $\mathbf{l}$, $\tilde{\mathbf{l}}$, C, $\tilde{C}$, T, $\tilde{T}$, 0)
12: CalculateXCovariancesA(n_{Q}, n_{S}, n_{ext}, $\mathbf{l}$, $\tilde{\mathbf{l}}$)
13: CalculateTransformations(n_{Q}, n_{S}, n_{ext}, $\mathbf{l}$, $\tilde{\mathbf{l}}$, 0)
14: CalculateApproxScores(n_{Q}, n_{S}, n_{ext}, $\mathbf{l}$, $\tilde{\mathbf{l}}$)
15: GetTopNTransformations(n_{Q}, n_{S}, n_{ext}, n_{tfm}, 0)
16: Calculate TM-scores [4] in parallel $\forall\, 0 \le q < n_{\mathrm{Q}}$, $0 \le s < n_{\mathrm{S}}$, and TF matrices $0 \le i < n_{\mathrm{tfm}}$ using the COMER2 DP algorithm [5]
17: GetTopNTransformations(n_{Q}, n_{S}, n_{tfm}, n_{brn}, 1)
18: OptimizeSelectedAlignments(n_{Q}, n_{S}, n_{brn})
19: RefineBestAlignments(n_{Q}, n_{S}, -0.6)
20: RefineBestAlignments(n_{Q}, n_{S}, 0)
21: **end procedure**

The post-processing phase (line 14) of order-independent alignments involves calculating approximate order-dependent TM-scores in sublinear time. A small number (n_{tfm}) of transformation matrices with the highest approximate scores are selected (line 15) to calculate TM-scores using the COMER2 DP algorithm

[5]. Requiring no backtracking information in line 16 reduces memory demands and increases the parallelization degree. A further refinement is made by selecting (line 17) an even smaller number ($n_{\mathrm{brn}} < n_{\mathrm{tfm}}$) of transformation matrices corresponding to the highest TM-scores to optimize structural alignments derived from them (line 18). Finally, the alignments—each representing one query-subject pair—with the highest TM-score are refined (lines 19–20).

Algorithm 2 Calculate Local Similarity

procedure CalculateLocalSimilarity(n_{Q}, n_{S}, n_{ext}, $\mathbf{l}$, $\tilde{\mathbf{l}}$, l_{f}, L, c_{thr})
  for all $(q, s, f_{\mathrm{ext}}) \in [0, n_{\mathrm{Q}}) \times [0, n_{\mathrm{S}}) \times [0, n_{\mathrm{ext}})$ **do in parallel**
    Calculate query and subject protein positions p_q and $\tilde{p}_s$ from index f_{ext}
    if $p_q + l_{\mathrm{f}} > l_q$ **or** $\tilde{p}_s + l_{\mathrm{f}} > \tilde{l}_s$ **then**
      Set *skip* flag for configuration $\{q, s, f_{\mathrm{ext}}\}$
      return
    end if
    $S \leftarrow 0_{32,32}$
    for all $i \in [0, \min\{96, l_q - p_q\})$ **do in parallel**
      for all $j \in [0, \min\{128, \tilde{l}_s - \tilde{p}_s\})$ **do in parallel**
        $[\ S_{i \bmod 32, j \bmod 32} \leftarrow \max\{S_{i \bmod 32, j \bmod 32}, L_{q,s,p_q+i,\tilde{p}_s+j}\}\ ]_{\mathrm{mut}}$
      end for
    end for
    $m \leftarrow \max_{i,j} S_{ij}$ ▷ two-dimensional parallel reduction
    if $m < l_{\mathrm{f}} \times c_{\mathrm{thr}}$ **then** ▷ fragment length fraction as a similarity threshold
      Set *skip* flag for configuration $\{q, s, f_{\mathrm{ext}}\}$
    end if
  end for
end procedure

Algorithm 3 Calculate cross-covariance matrices

procedure CalculateXCovariances(n_{Q}, n_{S}, n_{ext}, l_{f}, C, $\tilde{C}$)
  for all $(q, s, f_{\mathrm{ext}}) \in [0, n_{\mathrm{Q}}) \times [0, n_{\mathrm{S}}) \times [0, n_{\mathrm{ext}})$ **do in parallel**
    continue if *skip* flag is set for configuration $\{q, s, f_{\mathrm{ext}}\}$
    Calculate query and subject protein positions p_q and $\tilde{p}_s$ from index f_{ext}
    $(K_{qsf_{\mathrm{ext}}}, \mathbf{c}_{qf_{\mathrm{ext}}}, \tilde{\mathbf{c}}_{sf_{\mathrm{ext}}})$
      $\leftarrow \sum_{i=0}^{l_{\mathrm{f}}-1} (C_{q,p_q+i} \tilde{C}^{\mathrm{T}}_{s,\tilde{p}_s+i}, C_{q,p_q+i}, \tilde{C}_{s,\tilde{p}_s+i})$ ▷ parallel sum reduction
    Store $(K_{qsf_{\mathrm{ext}}}, \mathbf{c}_{qf_{\mathrm{ext}}}, \tilde{\mathbf{c}}_{sf_{\mathrm{ext}}})$ in memory
  end for
end procedure

Algorithm 4 Calculate transformation matrices

procedure CalculateTransformations(n_{Q}, n_{S}, n_{ext}, $\mathbf{l}$, $\tilde{\mathbf{l}}$, b_{dyn})
  for all $(q, s, f_{\text{ext}}) \in [0, n_{\text{Q}}) \times [0, n_{\text{S}}) \times [0, n_{\text{ext}})$ **do in parallel**
    continue if *skip* flag is set for configuration $\{q, s, f_{\text{ext}}\}$
    Load $(K_{qsf_{\text{ext}}}, \mathbf{c}_{qf_{\text{ext}}}, \tilde{\mathbf{c}}_{sf_{\text{ext}}})$ from memory
    if $b_{\text{dyn}} = 1$ **and** $l_q \geq \tilde{l}_s$ **then**
      $(K_{qsf_{\text{ext}}}, \mathbf{c}_{qf_{\text{ext}}}, \tilde{\mathbf{c}}_{sf_{\text{ext}}}) \leftarrow (K^{\text{T}}_{qsf_{\text{ext}}}, \tilde{\mathbf{c}}_{sf_{\text{ext}}}, \mathbf{c}_{qf_{\text{ext}}})$
    end if
    Calculate $R_{qsf_{\text{ext}}}$ by the Kabsch algorithm [6, 7]
      based on $(K_{qsf_{\text{ext}}}, \mathbf{c}_{qf_{\text{ext}}}, \tilde{\mathbf{c}}_{sf_{\text{ext}}})$
    $\mathbf{t}_{qsf_{\text{ext}}} \leftarrow \bar{\tilde{\mathbf{c}}}_{sf_{\text{ext}}} - R_{qsf_{\text{ext}}} \bar{\mathbf{c}}_{qf_{\text{ext}}}$
    Store $(R_{qsf_{\text{ext}}}, \mathbf{t}_{qsf_{\text{ext}}})$ in memory
  end for
end procedure

Algorithm 5 Produce pilot alignments in constant time using spatial indices

procedure AlignInConstantTime(n_{Q}, n_{S}, n_{ext}, $\mathbf{l}$, $\tilde{\mathbf{l}}$, C, $\tilde{C}$, T, $\tilde{T}$, b_{SSM})
  for all $(q, s, f_{\text{ext}}) \in [0, n_{\text{Q}}) \times [0, n_{\text{S}}) \times [0, n_{\text{ext}})$ **do in parallel**
    continue if *skip* flag is set for configuration $\{q, s, f_{\text{ext}}\}$
    Calculate query and subject protein positions p_q and $\tilde{p}_s$ from index f_{ext}
    $l_{\text{f}} \leftarrow \min\{512, l_q, \tilde{l}_s\}$
    if $l_q \geq \tilde{l}_s$ **then** ▷ always search in the larger structure
      $r \leftarrow \max\{0, \min\{\tilde{l}_s - l_{\text{f}}, \tilde{p}_s - l_{\text{f}}/2\}\}$
      $D \leftarrow \tilde{C}_{s,r:r+l_{\text{f}}-1}$; $\tilde{D} \leftarrow C_q$; $\theta \leftarrow \tilde{T}_{s,r:r+l_{\text{f}}-1}$
    else
      $r \leftarrow \max\{0, \min\{l_q - l_{\text{f}}, p_q - l_{\text{f}}/2\}\}$
      $D \leftarrow C_{q,r:r+l_{\text{f}}-1}$; $\tilde{D} \leftarrow \tilde{C}_s$; $\theta \leftarrow T_{q,r:r+l_{\text{f}}-1}$
    end if
    Load $(R_{qsf_{\text{ext}}}, \mathbf{t}_{qsf_{\text{ext}}})$ from memory
    for all $i \in [0, l_{\text{f}})$ **do in parallel**
      $D'_i \leftarrow R_{qsf_{\text{ext}}} D_i + \mathbf{t}_{qsf_{\text{ext}}}$ ▷ transformation
      $j \leftarrow$ nearest neighbour in $\tilde{D}$ for D'_i using index
        with ($b_{\text{SSM}} = 1$) or without ($b_{\text{SSM}} = 0$) information θ_i
        ▷ $O(1)$ time complexity
      Swap(D_i, $\tilde{D}_j$) **if** $l_q \geq \tilde{l}_s$
      Store $(D_i, \tilde{D}_j, j)$ in memory indexed by $(q, s, f_{\text{ext}}, i)$
    end for
  end for
end procedure

Algorithm 6 Calculate cross-covariance matrices from alignments

procedure CalculateXCovariancesA(n_{Q}, n_{S}, n_{ext}, $\mathbf{l}$, $\tilde{\mathbf{l}}$)
  for all $(q, s, f_{\text{ext}}) \in [0, n_{\text{Q}}) \times [0, n_{\text{S}}) \times [0, n_{\text{ext}})$ **do in parallel**
    continue if *skip* flag is set for configuration $\{q, s, f_{\text{ext}}\}$
    $l_{\text{f}} \leftarrow \min\{512, l_q, \tilde{l}_s\}$
    Load $(D_i, \tilde{D}_i, \cdot)_{i=0}^{l_{\text{f}}-1}$ from memory at $(q, s, f_{\text{ext}}, i)_{i=0}^{l_{\text{f}}-1}$
    $(K_{qsf_{\text{ext}}}, \mathbf{c}_{qf_{\text{ext}}}, \tilde{\mathbf{c}}_{sf_{\text{ext}}}) \leftarrow \sum_{i=0}^{l_{\text{f}}-1} (D_i \tilde{D}_i^{\text{T}}, D_i, \tilde{D}_i)$ ▷ parallel sum reduction
    Store $(K_{qsf_{\text{ext}}}, \mathbf{c}_{qf_{\text{ext}}}, \tilde{\mathbf{c}}_{sf_{\text{ext}}})$ in memory
  end for
end procedure

Algorithm 7 Calculate approximate order-dependent scores

procedure CalculateApproxScores(n_Q, n_S, n_ext, $\mathbf{l}$, $\tilde{\mathbf{l}}$)
  for all $(q, s, f_\mathrm{ext}) \in [0, n_\mathrm{Q}) \times [0, n_\mathrm{S}) \times [0, n_\mathrm{ext})$ **do in parallel**
    continue if *skip* flag is set for configuration $\{q, s, f_\mathrm{ext}\}$
    $l_\mathrm{f} \leftarrow \min\{512, l_q, \tilde{l}_s\}$
    $\mathbf{x} \leftarrow -\mathbf{1}_{512}$; $\mathbf{m} \leftarrow \mathbf{0}_{512}$
    Load $(R_{qsf_\mathrm{ext}}, \mathbf{t}_{qsf_\mathrm{ext}})$ from memory
    for all $i \in [0, l_\mathrm{f})$ **do in parallel**
      Load $(D_i, \tilde{D}_i, j)$ from memory at $(q, s, f_\mathrm{ext}, i)$
      $D'_i \leftarrow R_{qsf_\mathrm{ext}} D_i + \mathbf{t}_{qsf_\mathrm{ext}}$ ▷ transformation
      $z_i \leftarrow j$; $a_i \leftarrow d_0^2/(d_0^2 + \|D'_i - \tilde{D}_i\|^2)$ ▷ d_0 defined as in [4]
    end for
    for $i = 0, \ldots, l_\mathrm{f} - 1$ **do**
      $\omega \leftarrow \max_{j: x_j < z_i} m_j$ ▷ parallel max reduction
      $c \leftarrow z_i \bmod 512$ ▷ trivial hash function
      if $x_c < 0$ **or** $(x_c = z_i$ **and** $m_c < \omega + a_i)$ **or**
        $((i > l_\mathrm{f}/2)?\ x_c < z_i \colon x_c > z_i)$ ▷ heuristics upon hash collision
       then
        $x_c \leftarrow z_i$; $m_c \leftarrow \omega + a_i$
      end if
    end for
    $w_{qsf_\mathrm{ext}} \leftarrow \max_j m_j$ ▷ parallel reduction
    Store w_{qsf_ext} in memory
  end for
end procedure

Algorithm 8 Select n_tfm top-performing transformation matrices

procedure GetTopNTransformations(n_Q, n_S, n_ext, n_tfm, b_srt)
  for all $(q, s) \in [0, n_\mathrm{Q}) \times [0, n_\mathrm{S})$ **do in parallel**
    $\mathbf{x} \leftarrow -\mathbf{1}_{n_\mathrm{tfm}}$; $\mathbf{m} \leftarrow \mathbf{0}_{n_\mathrm{tfm}}$
    if $b_\mathrm{srt} = 1$ **then**
      Load $(w_{qsf_\mathrm{ext}})_{f_\mathrm{ext}=0}^{n_\mathrm{ext}-1}$ from memory ▷ TM-scores if obtained by DP
      $\mathbf{x}' \leftarrow$ Sort$((w_{qsf_\mathrm{ext}})_{f_\mathrm{ext}})$ ▷ Batcher's sort [8] in $O(\log_2^2 n_\mathrm{ext})$ time
      $\mathbf{x} \leftarrow (x'_i)_{i=0}^{n_\mathrm{tfm}-1}$ ▷ $x_i = -1$ if $w_{qsx'_i} = 0$ (*skip* flag set)
    else
      for all $(f_\mathrm{ext}) \in [0, n_\mathrm{ext})$ **do in parallel** ▷ approx. to partial sorting
        continue if *skip* flag is set for configuration $\{q, s, f_\mathrm{ext}\}$
        Load w_{qsf_ext} from memory
        $f_\mathrm{m} \leftarrow f_\mathrm{ext} \bmod n_\mathrm{tfm}$
        $[\,(m_{f_\mathrm{m}}, x_{f_\mathrm{m}}) \leftarrow (w_{qsf_\mathrm{ext}}, f_\mathrm{ext})$ **if** $m_{f_\mathrm{m}} < w_{qsf_\mathrm{ext}}\,]_\mathrm{mut}$
      end for
    end if
    for all $i \in [0, n_\mathrm{tfm})$ **do in parallel**
      Set $(x_i < 0)$ or unset $(x_i \geq 0)$ *skip* flag for configuration $\{q, s, i\}$
      if $x_i \geq 0$ **then**
        Load $(R_{qsx_i}, \mathbf{t}_{qsx_i})$ from memory
        $(R'_{qsi}, \mathbf{t}'_{qsi}) \leftarrow (R_{qsx_i}, \mathbf{t}_{qsx_i})$
      end if
    end for
    for all $i \in [0, n_\mathrm{tfm})$ **do in parallel**
      if $x_i \geq 0$ **then**
        $(R_{qsi}, \mathbf{t}_{qsi}) \leftarrow (R'_{qsi}, \mathbf{t}'_{qsi})$
        Store $(R_{qsi}, \mathbf{t}_{qsi})$ in memory
      end if
    end for
  end for
end procedure

Algorithm 9 Optimize n_{brn} selected alignments

```
procedure OptimizeSelectedAlignments(n_Q, n_S, n_brn)
    for i = 0, ..., n_brn − 1 do
        for all (q, s) ∈ [0, n_Q) × [0, n_S) do in parallel
            continue if skip flag is set for configuration {q, s, i}
            Load TF matrix (R_qsi, t_qsi) from memory
            Perform DP using (R_qsi, t_qsi) and the COMER2 DP algorithm [5]
            Produce alignment using the COMER2 backtracking algorithm
            Store l_A(R_qsi, t_qsi) aligned residues' coordinates (C_qk, C̃_sl)_k,l in memory
            Find optimal (R_qsi, t_qsi) by calculating TM-scores
                based on the superpositions obtained in parallel from
                n_A[l_A(R_qsi, t_qsi)] different-length and differently positioned
                alignment fragments (similarly to "Search Engine" in [4])
        end for
    end for
    for all (q, s) ∈ [0, n_Q) × [0, n_S) do in parallel
        Store (R_qs, t_qs)
            ≡ argmax_{(R_qsi, t_qsi)}_{i=0}^{n_brn−1} TM-score(R_qsi, t_qsi) in memory
    end for
end procedure
```

Algorithm 10 Refine best alignments

```
procedure RefineBestAlignments(n_Q, n_S, c_goc)
    repeat 2 times
        for all (q, s) ∈ [0, n_Q) × [0, n_S) do in parallel
            Load TF matrix (R_qs, t_qs) from memory
            Perform DP using (R_qs, t_qs) and the COMER2 DP algorithm [5]
                with gap open cost c_goc
            Produce alignment using the COMER2 backtracking algorithm
            Store l_A(R_qs, t_qs) aligned residues' coordinates (C_qk, C̃_sl)_k,l in memory
            Find (R'_qs, t'_qs) that maximizes TM-score
                based on the superpositions obtained in parallel from
                n_A[l_A(R_qs, t_qs)] different-length and differently positioned
                alignment fragments (similarly to "Search Engine" in [4])
            Store (R_qs, t_qs) = (R'_qs, t'_qs) in memory
        end for
    end
end procedure
```

Bibliography

[1] Söding, J. & Remmert, M. Protein sequence comparison and fold recognition: progress and good-practice benchmarking. *Curr. Opin. Struct. Biol.* **21**, 404–411 (2011).

[2] Burley, S. *et al.* RCSB Protein Data Bank: powerful new tools for exploring 3d structures of biological macromolecules for basic and applied research and education in fundamental biology, biomedicine, biotechnology, bioengineering and energy sciences. *Nucleic Acids Res.* **49**, D437–D451 (2020).

[3] Defays, D. An efficient algorithm for a complete link method. *Comput. J.* **20**, 364–366 (1977).

[4] Zhang, Y. & Skolnick, J. Scoring function for automated assessment of protein structure template quality. *Proteins* **57**, 702–710 (2004).

[5] Margelevičius, M. COMER2: GPU-accelerated sensitive and specific homology searches. *Bioinformatics* **36**, 3570–3572 (2020).

[6] Kabsch, W. A solution for the best rotation to relate two sets of vectors. *Acta Crystallogr. A* **32**, 922–923 (1976).

[7] Kabsch, W. A discussion of the solution for the best rotation to relate two sets of vectors. *Acta Crystallogr. A* **34**, 827–828 (1978).

[8] Batcher, K. E. Sorting networks and their applications. *Proceedings of the April 30–May 2, 1968, Spring Joint Computer Conference* 307–314 (1968).
