# Supplementary material for: GTalign: spatial index-driven protein structure alignment, superposition, and search
Source: Nat Commun. 2024 Aug 24;15:7305. doi: 10.1038/s41467-024-51669-z (PMC11344802; doi:10.1038/s41467-024-51669-z)
Supplement: Supplementary file 3 — Reporting Summary [file 41467_2024_51669_MOESM3_ESM.pdf]

nature portfolio

Corresponding author(s): Mindaugas Margelevicius

Last updated by author(s): Aug 5, 2024

Reporting Summary

Nature Portfolio wishes to improve the reproducibility of the work that we publish. This form provides structure for consistency and transparency in reporting. For further information on Nature Portfolio policies, see our Editorial Policies and the Editorial Policy Checklist.

Statistics

For all statistical analyses, confirm that the following items are present in the figure legend, table legend, main text, or Methods section.

n/a	Confirmed	
☐	☒	The exact sample size (*n*) for each experimental group/condition, given as a discrete number and unit of measurement
☐	☒	A statement on whether measurements were taken from distinct samples or whether the same sample was measured repeatedly
☐	☒	The statistical test(s) used AND whether they are one- or two-sided<br>*Only common tests should be described solely by name; describe more complex techniques in the Methods section.*
☒	☐	A description of all covariates tested
☒	☐	A description of any assumptions or corrections, such as tests of normality and adjustment for multiple comparisons
☐	☒	A full description of the statistical parameters including central tendency (e.g. means) or other basic estimates (e.g. regression coefficient) AND variation (e.g. standard deviation) or associated estimates of uncertainty (e.g. confidence intervals)
☐	☒	For null hypothesis testing, the test statistic (e.g. F, t, r) with confidence intervals, effect sizes, degrees of freedom and P value noted<br>*Give P values as exact values whenever suitable.*
☒	☐	For Bayesian analysis, information on the choice of priors and Markov chain Monte Carlo settings
☒	☐	For hierarchical and complex designs, identification of the appropriate level for tests and full reporting of outcomes
☒	☐	Estimates of effect sizes (e.g. Cohen's *d*, Pearson's *r*), indicating how they were calculated

Our web collection on statistics for biologists contains articles on many of the points above.

Software and code

Policy information about availability of computer code

Data collection	No software was used.
Data analysis	GTalign version 0.14.0 and 0.15.0, the method introduced: https://github.com/minmarg/gtalign_alpha (Github repository), https://zenodo.org/records/10433420 (version 0.14.0; doi: 10.5281/zenodo.10433420), https://zenodo.org/records/10433419 (versions >0.14.0; doi: 10.5281/zenodo.10433419).<br>Other protein structure alignment tools: TM-align version 20220412; standalone version DaliLite.v5; DeepAlign version v1.4 Aug-20-2018; FATCAT version 2.0; Foldseek version d1d1b868a571a9a0c62ae50b07139ebdd224f879 (downloaded 6/25/2023).<br>Software for sequence clustering: blastclust version 2.2.26.<br>Molecular graphics software for visualizations: UCSF Chimera version 1.14.<br>Software for creating plots and statistical tests: R versions 3.6.0 and 4.3.2.

For manuscripts utilizing custom algorithms or software that are central to the research but not yet described in published literature, software must be made available to editors and reviewers. We strongly encourage code deposition in a community repository (e.g. GitHub). See the Nature Portfolio guidelines for submitting code & software for further information.

Data

Policy information about availability of data

All manuscripts must include a data availability statement. This statement should provide the following information, where applicable:

- Accession codes, unique identifiers, or web links for publicly available datasets
- A description of any restrictions on data availability
- For clinical datasets or third party data, please ensure that the statement adheres to our policy

The SCOPe 2.08 PDB-style files with coordinates for the SCOPe40 2.08 dataset are available at https://scop.berkeley.edu/downloads/. The PDB files for the PDB20 dataset are available at https://www.rcsb.org/downloads. The archive of the Swiss-Prot protein structures is available at https://ftp.ebi.ac.uk/pub/databases/alphafold/latest/swissprot_pdb_v4.tar. The HOMSTRAD dataset, originally obtained from http://yanglab.nankai.edu.cn/mTM-align/benchmark, is available at https://github.com/minmarg/gtalign-evaluation. The benchmark data generated in this study have been deposited in the Zenodo database under accession code 10.5281/zenodo.11148017 (https://doi.org/10.5281/zenodo.11148017). Source data are provided with this paper.

Research involving human participants, their data, or biological material

Policy information about studies with human participants or human data. See also policy information about sex, gender (identity/presentation), and sexual orientation and race, ethnicity and racism.

Reporting on sex and gender	This information has not been collected.
Reporting on race, ethnicity, or other socially relevant groupings	n/a
Population characteristics	n/a
Recruitment	n/a
Ethics oversight	n/a

Note that full information on the approval of the study protocol must also be provided in the manuscript.

Field-specific reporting

Please select the one below that is the best fit for your research. If you are not sure, read the appropriate sections before making your selection.

☒ Life sciences ☐ Behavioural & social sciences ☐ Ecological, evolutionary & environmental sciences

For a reference copy of the document with all sections, see nature.com/documents/nr-reporting-summary-flat.pdf

Life sciences study design

All studies must disclose on these points even when the disclosure is negative.

Sample size	Evaluations were conducted across four diverse datasets: SCOPe 2.08 protein domains filtered to 40% sequence identity (15,177 protein structures), PDB full-length structures filtered to 20% sequence identity (18,801 structures), the UniProtKB/Swiss-Prot protein structures from the AlphaFold Database (542,378 structures), and the HOMSTRAD database (398 multiple protein structure alignments). All data for these datasets were obtained from the respective websites. The four widely used large-scale datasets provide reliable performance estimates.
Data exclusions	The PDB20 dataset was filtered using the blastclust tool to make a highly diverse set of proteins.
Replication	All software settings are specified to reproduce the results. The benchmark scripts and commands are provided.
Randomization	Complete datasets were used to benchmark protein structure alignment tools. For the SCOPe 2.08 dataset, 2045 queries, one per superfamily, were selected randomly. For the Swiss-Prot dataset, to increase structural diversity, 40 queries related to CRISPR-Cas systems were randomly selected from the PDB.
Blinding	Blinding is not relevant to benchmarking protein structure alignment tools, as either complete datasets or randomly selected queries were used.

Reporting for specific materials, systems and methods

We require information from authors about some types of materials, experimental systems and methods used in many studies. Here, indicate whether each material, system or method listed is relevant to your study. If you are not sure if a list item applies to your research, read the appropriate section before selecting a response.

Materials & experimental systems

n/a	Involved in the study
☒	☐ Antibodies
☒	☐ Eukaryotic cell lines
☒	☐ Palaeontology and archaeology
☒	☐ Animals and other organisms
☒	☐ Clinical data
☒	☐ Dual use research of concern
☒	☐ Plants

Methods

n/a	Involved in the study
☒	☐ ChIP-seq
☒	☐ Flow cytometry
☒	☐ MRI-based neuroimaging

Plants

Seed stocks	n/a
Novel plant genotypes	n/a
Authentication	n/a
